# Impact Factor Trends of Top Obstetrics and Gynecology Journals During COVID-19

**DOI:** 10.2196/70554

**Published:** 2025-06-17

**Authors:** Minhazur Sarker, Emily Yang, Ukachi Emeruwa, Timothy Wen

**Affiliations:** 1 Division of Maternal Fetal Medicine, Department of Obstetrics, Gynecology, and Reproductive Sciences, University of California, San Diego, 9300 Campus Point Dr, Mail Code 7433, San Diego, CA, 92037, United States, 1 858 249 1207; 2 Department of Obstetrics, Gynecology, and Reproductive Sciences, University of California, San Diego, San Diego, CA, United States; 3 Department of Medicine, University of California, San Diego, San Diego, CA, United States

**Keywords:** impact factor, COVID-19, research impact

## Abstract

Obstetrics and gynecology journal impact factor trends during the COVID-19 pandemic were similar to those seen among other medical specialties, and our findings further highlight the ongoing need to implement a metric of research impact that is not as easily manipulated by selective publication.

## Introduction

The impact factor (IF), originally introduced as a metric to aid librarian purchasing decisions, has quickly become a marker for journal prestige [1]. Over the years, the concept of IFs has been highly criticized, as they are susceptible to manipulation and may be a disservice to science and their readers [1-4]. While temporal changes may provide insight into journal growth, IFs are also uniquely affected by publication trends. Studies have shown that the COVID-19 pandemic created an unprecedented surge of highly impactful publications increasing IFs [5-8]. Whether an IF uptrend was appreciated among obstetrics and gynecology (OBGYN) journals during COVID-19 and whether the changes reflect a COVID-19 “blockbuster” effect or genuine advancement in women’s health research remains unknown. We aimed to determine the IF trends of the top OBGYN journals and compare them to representative journals for other medical specialties (non-OBGYN).

## Methods

Using the SCImago Journal Rank database, we identified the top 30 broad scope women’s health journals (Multimedia Appendix 1). Non-OBGYN national societies were queried to highlight each subspecialty’s representative journal. Annual IFs were identified using a web-based searchable database, and temporal trends were assessed over three time periods: (1) prepandemic (2016‐2019), (2) COVID-19 pandemic (2019‐2021), and (3) postpandemic (2021‐2023) [9]. IF trends by individual journal and composite journal type were assessed using the National Cancer Institute’s Joinpoint Regression Program, with measures of association expressed with annual average percent changes (AAPCs) and 95% CIs [10]. The average IF percent changes between composite journal types were compared using Student *t* test. Institutional review board approval was not required for this study.

## Results

**Figure 1.**
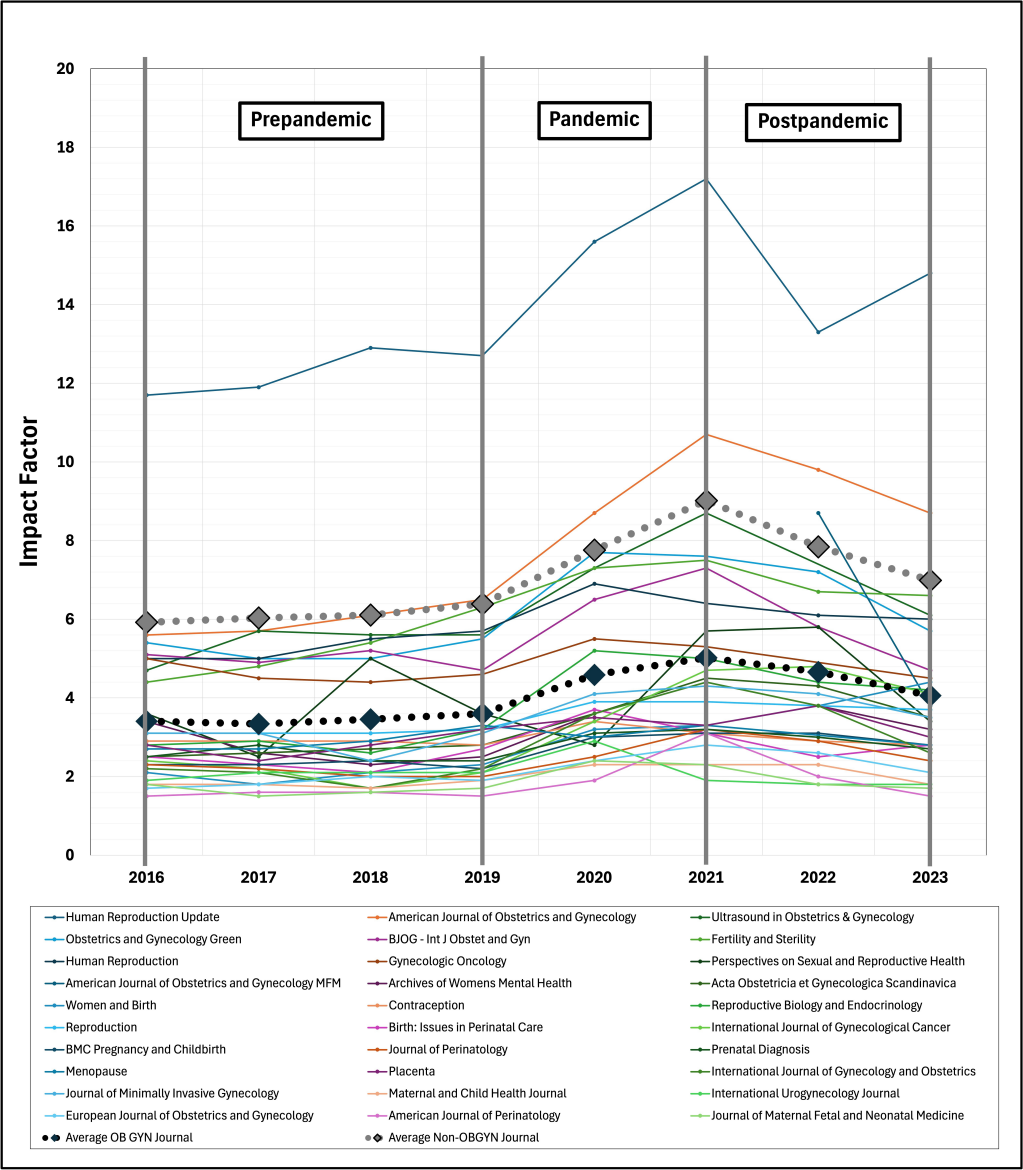
Impact factor trends among the top 30 OBGYN journals. The dotted black line represents the average impact factor among OBGYN journals, while the dotted gray line represents the average impact factor among non-OBGYN journals. OBGYN: obstetrics and gynecology.

IF data obtained from the top 30 OBGYN journals were compared to 19 non-OBGYN journals. From 2016 to 2023, the mean OBGYN journal IF increased from 3.4 to 4.1 (AAPC 4.1%, 95% CI 0.8%-4.1%) compared to 5.9 to 7.0 (AAPC 3.6%, 95% CI 0.4%-7.5%) for non-OBGYN journals with an average percent change of 18.8% compared to 16.3%, respectively (*P*=.91; Figures1  2).

**Figure 2.**
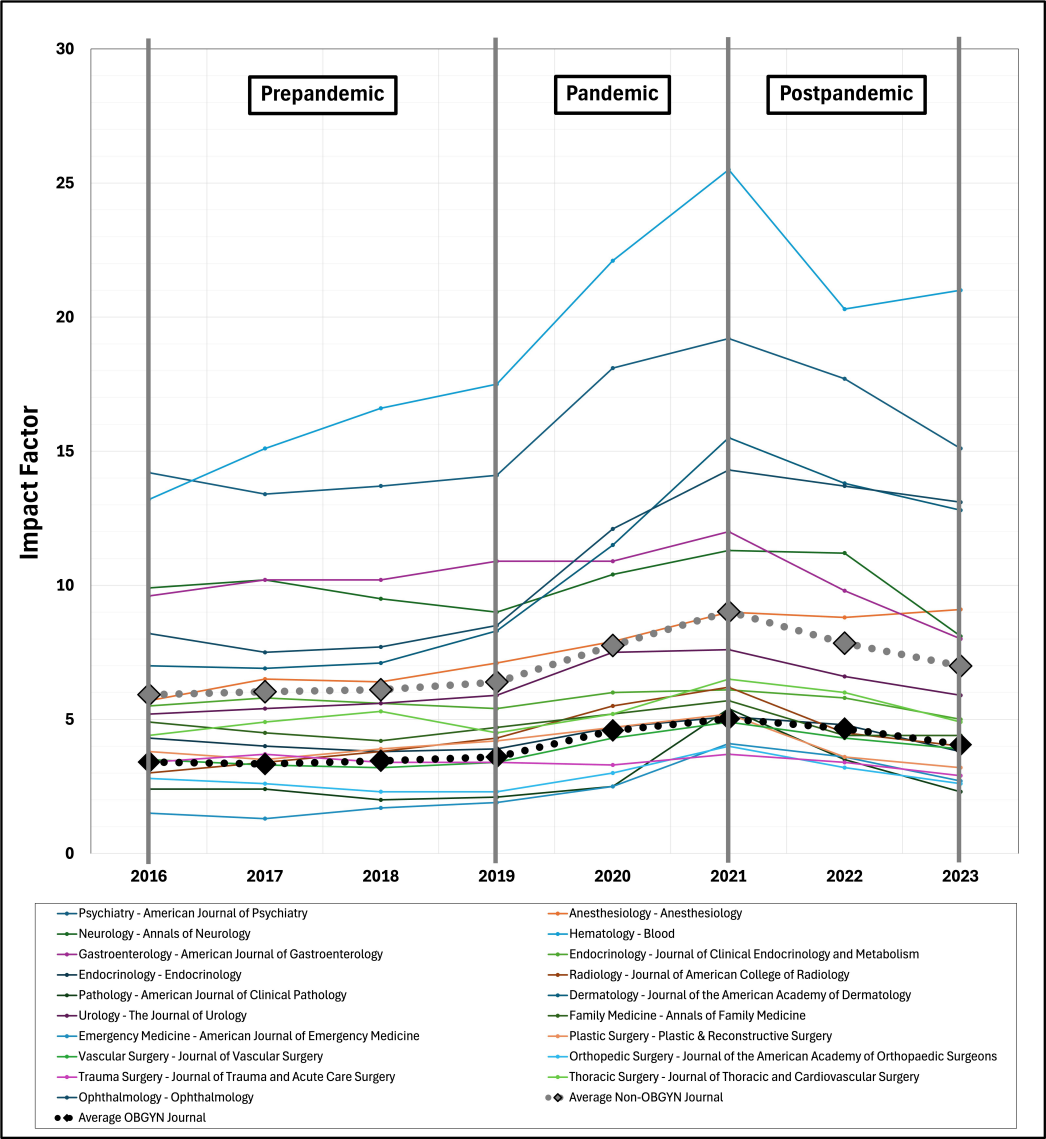
Impact factor trends among 19 non-OBGYN medical specialty-specific journals. The dotted black line represents the average impact factor among OBGYN journals, while the dotted gray line represents the average IF among non-OBGYN journals. OBGYN: obstetrics and gynecology.

In the prepandemic period, the mean OBGYN journal IF increased from 3.4 to 3.6 (AAPC 8.9%, 95% CI 4.7%-18.1%) compared to 5.9 to 6.4 (AAPC 8.8%, 95% CI 5.0%-17.8%) for non-OBGYN journals with an average percent change of 4.4% compared to 6.9%, respectively (*P*=.57). During the pandemic, the mean OBGYN journal IF increased from 3.6 to 5.0 (AAPC 8.9%, 95% CI –1.1% to 13.6%) compared to 6.4 to 9.0 (AAPC 8.8%, 95% CI –1.8% to 13.4%) for non-OBGYN journals with an average percent change of 42.7% compared to 47.6%, respectively (*P*=.63). Finally, in the postpandemic period, the mean OBGYN journal IF decreased from 5.0 to 4.1 (AAPC –7.2%, 95% CI –18.2% to 4.3%) compared to 9.0 to 7.0 (AAPC –8.3%, 95% CI –19.0% to 2.9%) for non-OBGYN journals with an average percent change of –18.3% compared to –25.7%, respectively (*P*=.16).

## Discussion

Our findings indicated significant increases in overall IF trends from 2016 to 2023 among OBGYN journals, though not statistically different from non-OBGYN journals. When analyzing prepandemic, during the pandemic, and postpandemic periods, both OBGYN and non-OBGYN journals exhibited a similar pattern of small increases prepandemic, robust uptrends during the pandemic, then a steep but less pronounced decline post pandemic (Figures1  2). Given these results, it is likely that IF uptrends were an artificial COVID-19–related “blockbuster” effect rather than genuine advancements in women’s health research.

The COVID-19 trends shown here among OBGYN journals have similarly been seen among medicine, global health, and infectious disease journals during the pandemic [5,6,8]. On one hand, the rapid surge in publications shows how strongly we can mobilize and disseminate research early and effectively to quickly improve health outcomes. On the other hand, there was pressure among journals to publish COVID-19 literature given its highly impactful and citable nature. Beyond being used for an indication it was not intended for, the IF has now repeatedly been highly criticized by many studies yet remains the most mainstream metric to discuss journal importance or prestige.

Our study has limitations. We are unable to comment on authors’ research funding changes or each journal’s editorial policies and shifts in publication practices during the pandemic. Additionally, our analysis was unable to adjust for journal size, scope, or publication frequency.

Using IFs alone in their current capacity may lead to wrongly concluding genuine research advancement in OBGYN. Among the public, this misunderstanding of the IF has the potential to create further distrust of medical literature. Our study further highlights the ongoing need to implement a different metric of research impact such as a composite using the IF, the *h*-index, and altmetrics that are not as easily manipulated by selective publication.

## Supplementary material

10.2196/70554 Multimedia Appendix 1 SCImago Journal Rank search strategy.

## Abbreviations

AAPC: annual average percent change
IF: impact factor
OBGYN: obstetrics and gynecology
