# Supplementary material for: Impact Factor Trends of Top Obstetrics and Gynecology Journals During COVID-19
Source: Interact J Med Res. 2025 Jun 17;14:e70554. doi: 10.2196/70554 (PMC12187028; doi:10.2196/70554)
Supplement: Multimedia Appendix 1 [file ijmr-v14-e70554-s001.docx]

1. Scimagojr online database was accessed via [www.scimagojr.com](http://www.scimagojr.com)


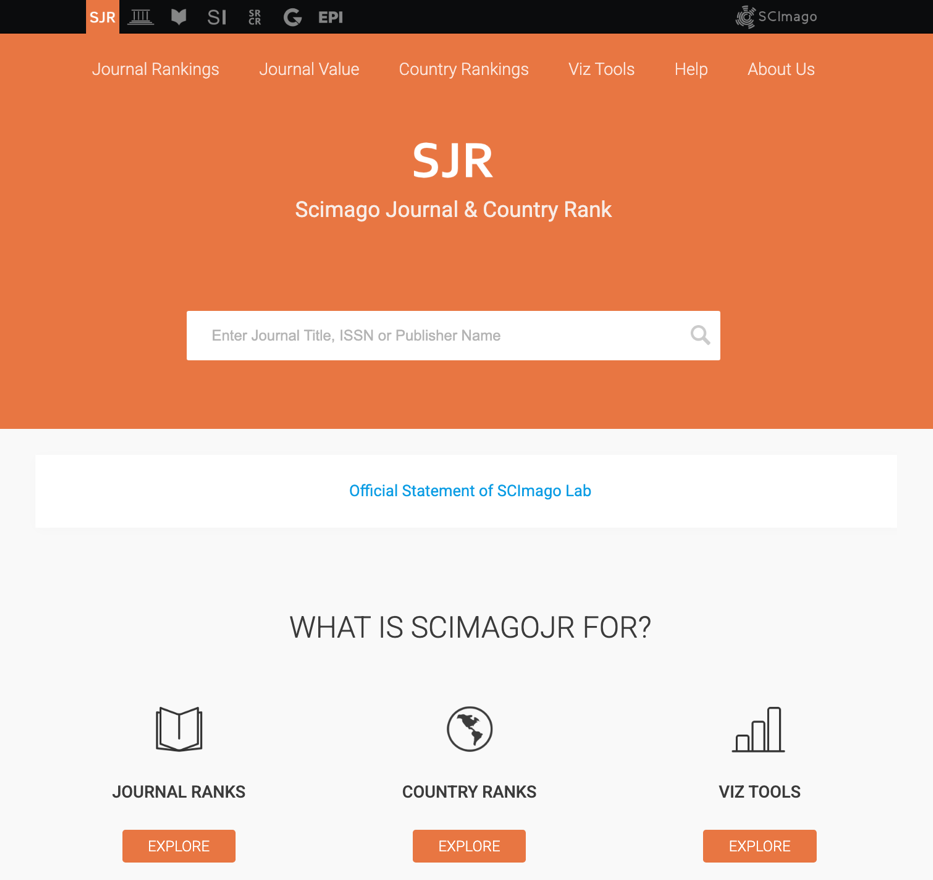


1. “Journal Rankings” was clicked next to open up journal rankings section.


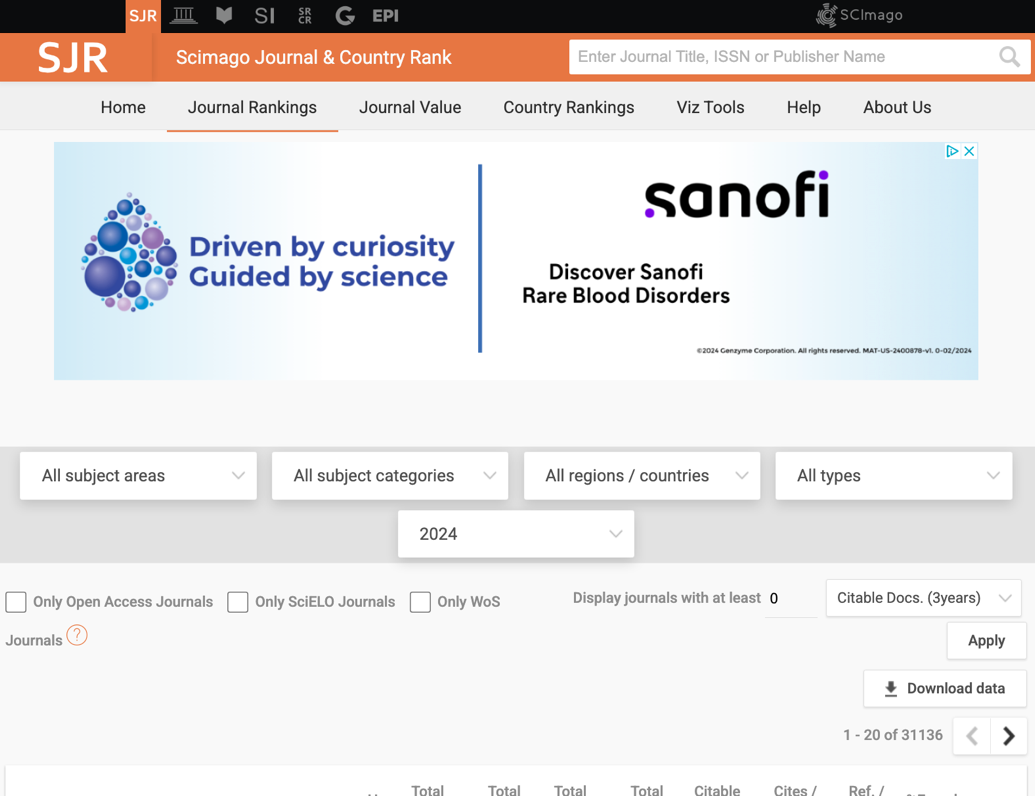


1. “Obstetrics and Gynecology” was selected from “all subject categories” filter.


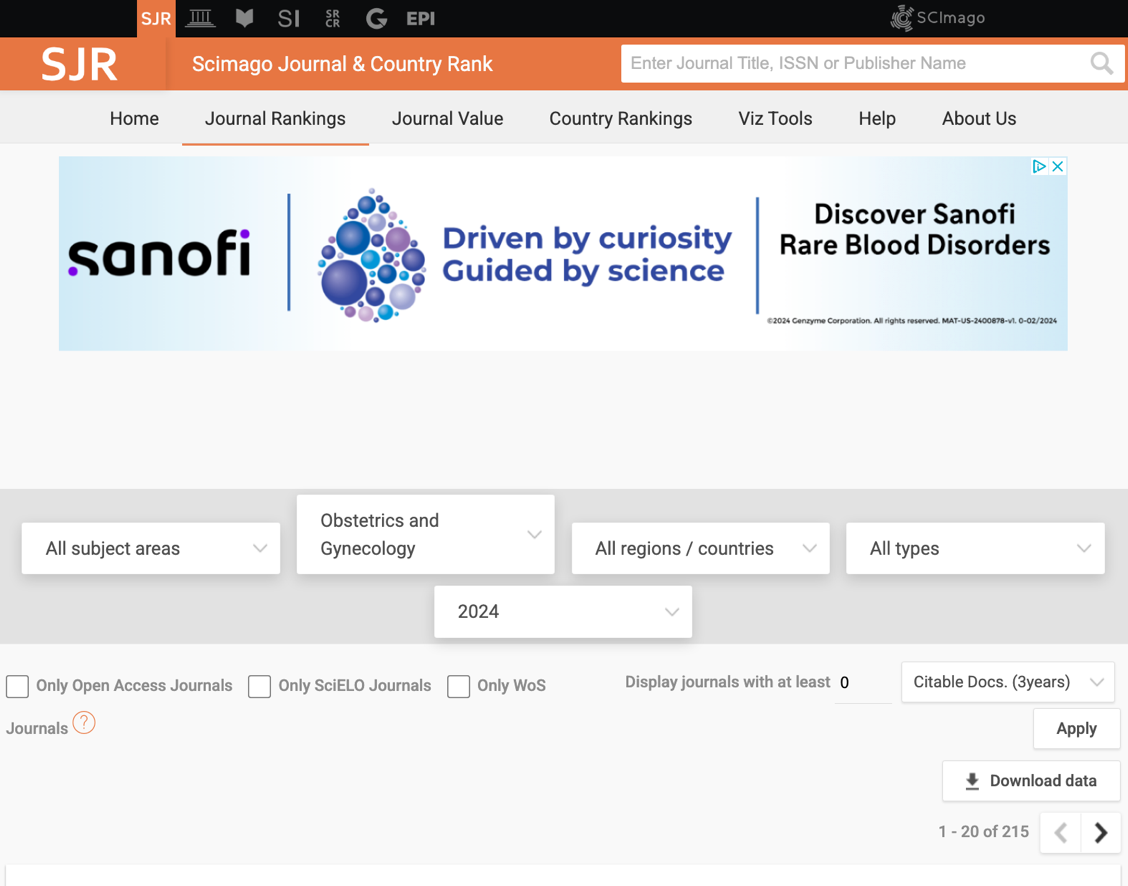


1. The returned search was then queried for the top 30 OB GYN journals published in English.


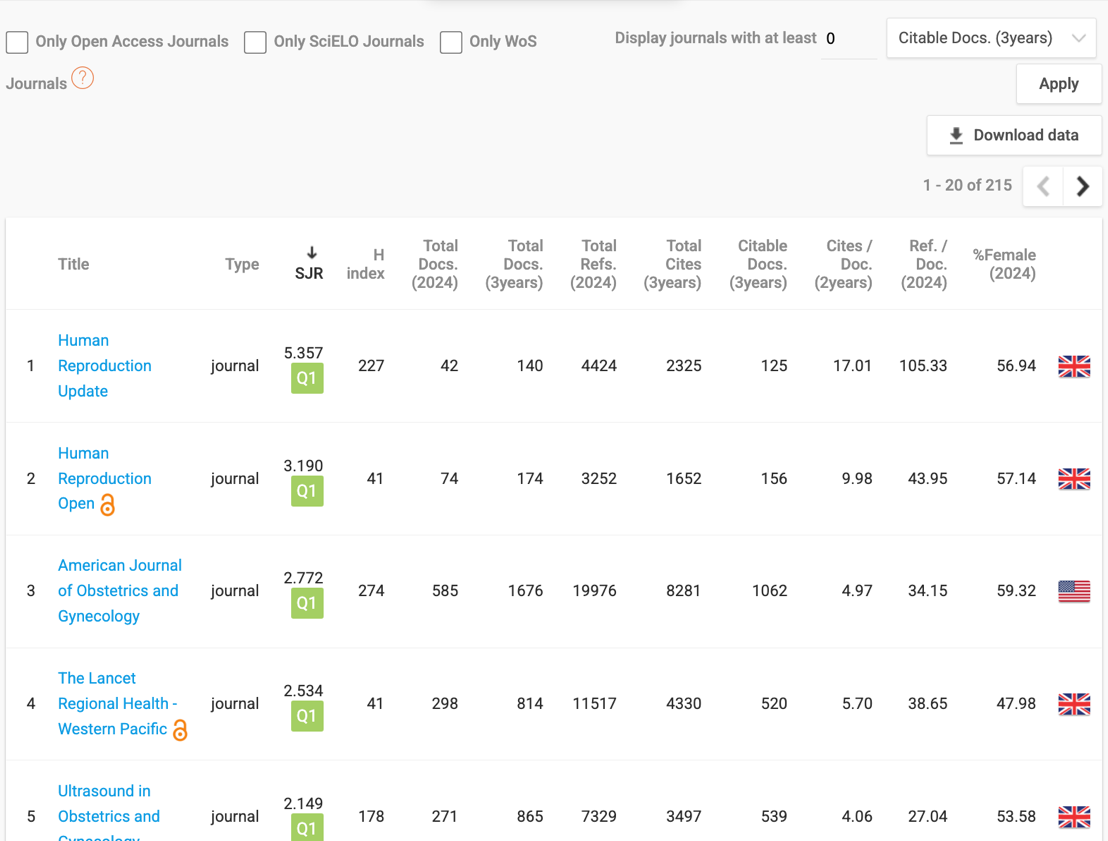


1. The targeted number of journals was set to 30 as after the first 30 English journals, there were significantly higher numbers of non-English journals and more targeted scope journals instead of broad women’s health journals.
